# Neurophysiological and Brain Structural Markers of Cognitive Frailty Differ from Alzheimer's Disease

**DOI:** 10.1523/JNEUROSCI.0697-21.2021

**Published:** 2022-02-16

**Authors:** Ece Kocagoncu, David Nesbitt, Tina Emery, Laura E. Hughes, Richard N. Henson, James B. Rowe, Cambridge Center for Ageing and Neuroscience

**Affiliations:** ^1^Department of Clinical Neurosciences, University of Cambridge, Cambridge, CB2 0SZ, United Kingdom; ^2^MRC Cognition and Brain Sciences Unit, University of Cambridge, Cambridge, CB2 7EF, United Kingdom; ^3^Department of Psychiatry, University of Cambridge, Cambridge, CB2 0SZ, United Kingdom; ^4^Cambridge University Hospital NHS Foundation Trust, University of Cambridge, Cambridge, CB2 2QQ, United Kingdom

**Keywords:** aging, Alzheimer's disease, cognitive frailty, cognitive reserve, MEG, MRI

## Abstract

With increasing life span and prevalence of dementia, it is important to understand the mechanisms of cognitive aging. Here, we focus on a subgroup of the population we term “cognitively frail,” defined by reduced cognitive function in the absence of subjective memory complaints, or a clinical diagnosis of dementia. Cognitive frailty is distinct from cognitive impairment caused by physical frailty. It has been proposed to be a precursor to Alzheimer's disease, but may alternatively represent one end of a nonpathologic spectrum of cognitive aging. We test these hypotheses in humans of both sexes, by comparing the structural and neurophysiological properties of a community-based cohort of cognitive frail adults, to people presenting clinically with diagnoses of Alzheimer's disease or mild cognitive impairment, and community-based cognitively typical older adults. Cognitive performance of the cognitively frail was similar to those with mild cognitive impairment. We used a novel cross-modal paired-associates task that presented images followed by sounds, to induce physiological responses of novelty and associative mismatch, recorded by EEG/MEG. Both controls and cognitively frail showed stronger mismatch responses and larger temporal gray matter volume, compared with people with mild cognitive impairment and Alzheimer's disease. Our results suggest that community-based cognitively frail represents a spectrum of normal aging rather than incipient Alzheimer's disease, despite similar cognitive function. Lower lifelong cognitive reserve, hearing impairment, and cardiovascular comorbidities might contribute to the etiology of the cognitive frailty. Critically, community-based cohorts of older adults with low cognitive performance should not be interpreted as representing undiagnosed Alzheimer's disease.

**SIGNIFICANCE STATEMENT** The current study investigates the neural signatures of cognitive frailty in relation to healthy aging and Alzheimer's disease. We focus on the cognitive aspect of frailty and show that, despite performing similarly to the patients with mild cognitive impairment, a cohort of community-based adults with poor cognitive performance do not show structural atrophy or neurophysiological signatures of Alzheimer's disease. Our results call for caution before assuming that cognitive frailty represents latent Alzheimer's disease. Instead, the cognitive underperformance of cognitively frail adults could result in cumulative effects of multiple psychosocial risk factors over the lifespan, and medical comorbidities.

## Introduction

With longer life span and an older population, there is a pressing need to understand the mechanisms that determine cognitive aging, and its relationship to dementias. The cognitively frail is a population of interest, defined by reduced cognitive function in the absence of subjective memory complaints, or a clinical diagnosis of dementia, mild cognitive impairment (MCI), or other preexisting neurologic explanation. Here cognitive frailty does not refer only to the cognitive impairments of those with comorbid physical frailty (Kelaiditi et al., 2013). Cognitive frailty has been linked to a higher risk of dementia, and is often regarded as a precursor to Alzheimer's disease (Panza et al., 2006; Buchman et al., 2007; Kojima et al., 2016; Shimada et al., 2018). In the absence of physical frailty, cognitive impairment alone is associated with longitudinal decline in functional abilities, activities of daily living (Shimada et al., 2016), increased hospitalization, and all-cause mortality rate (Avila-Funes et al., 2012; Solfrizzi et al., 2012; Ge et al., 2020).

However, there is an alternative hypothesis: poor cognitive performance reflects adverse aspects of normal aging, without latent Alzheimer's disease or other neurodegenerative process. Psychosocial, educational, medical factors may contribute to cognitive frailty in the absence of latent degenerative or vascular dementia pathologies. For example, cognitively underperforming adults are 4 times more likely to come from disadvantaged socioeconomic backgrounds, and twice as likely to have lower educational qualifications (Rogers et al., 2017). They are more likely to be malnourished (Mulero et al., 2011; Talegawkar et al., 2012; Chye et al., 2018), have a sedentary lifestyle (Landi et al., 2010; Rogers et al., 2017), and have more medical comorbidities, such as cardiovascular disease (Patrick et al., 2002; Langlois et al., 2012; Fuhrmann et al., 2019), chronic inflammation (Walston et al., 2002; Weaver et al., 2002; Cappola et al., 2003), and hearing impairment (Valentijn et al., 2005; Panza et al., 2015).

Here we determine whether cognitively frail community dwelling older adults have structural and/or neurophysiological characteristics of normal aging or early Alzheimer's disease. We quantify brain structure and function using MRI and EEG/MEG respectively. In Alzheimer's disease, amyloid plaques and neurofibrillary tangles form early in entorhinal cortex and hippocampi (Hardy and Selkoe, 2002; Braak et al., 2006), leading to disruptions in synaptic and neural function (LaFerla and Oddo, 2005; West and Bhugra, 2015) and atrophy. If the cognitively frail have prodromal or undiagnosed Alzheimer's disease pathology, one would expect similar structural and neurophysiological changes. To assess the neural systems of hippocampal-dependent associative memory, we designed the cross-modal oddball task. The trials consisted of repeated pairings of an abstract image with a sound. A mismatch response arose from pairs that included either a novel sound (i.e., novelty deviant [DN]) or a sound that was not novel but had been previously associated with a different image (i.e., associative deviant [DA]). The DNs are akin to typical mismatch negativity responses, proposed to be an index of auditory predictive coding, which is attenuated in Alzheimer's disease (Ruzzoli et al., 2016; Laptinskaya et al., 2018). The DAs are a more sensitive test of Alzheimer's disease, since hippocampal dysfunction would impair the ability to establish cross-modal associations (Gottfried and Dolan, 2003; Joassin et al., 2011), and attenuate the response to DAs. Indeed, previous studies report impaired performance on the paired-associates learning task in MCI and preclinical Alzheimer's disease, which correlates with disease progression (Blackwell et al., 2004; Ahmed et al., 2008). Moreover, in fMRI, paired-associates learning task shows Alzheimer's-related increased hippocampal activity and connectivity between hippocampus and cortical areas (de Rover et al., 2011; Harrison et al., 2016). We measured DN and DA responses in lateral frontotemporal areas, readily detected by EEG/MEG in auditory oddball paradigms and reduced in dementia (Pekkonen, 2000; Garrido et al., 2009; Phillips et al., 2016).

We proposed that, if cognitively frailty represents part of the spectrum of normal aging, rather than latent Alzheimer's disease, then the neurophysiological responses and structural features of cognitively frail adults would resemble cognitively healthy adults rather than the patients with MCI or Alzheimer's disease.

## Materials and Methods

### Study design

The Cambridge Center for Ageing and Neuroscience (Cam-CAN) Frail Project is an extension of the large-scale cross-sectional population-based Cam-CAN (Shafto et al., 2014), focused on cognitive frailty. It examines the subpopulation of community-dwelling adults with cognitive frailty, identified from home screening visits by cognitive screening tests: below 25/30 on the Mini Mental State Examination (MMSE) and/or below 88/100 on the Addenbrooke's Cognitive Examination–Revised (ACE-R) in the absence of a diagnosis or referral for a memory disorder.

The Cam-CAN Frail protocol comprised of three sessions. First, a visit to the participant's home to assess lifestyle, health, and cognitive performance on an extensive neuropsychological test battery. The battery included the ACE-R, MMSE, Wechsler Adult Intelligence Scale logical memory test, Spot the Word test, simple choice reaction time, famous faces test, four-mountains task, virtual object location and orientation, Rey figure recall, and the trail making test. In the second session, participants underwent EEG/MEG scanning and completed the Cattell and digit symbol tests. During the EEG/MEG recording, participants completed the cross-modal oddball task. In the final session, participants had an fMRI and structural MRI and completed the Hotel task. The study was approved by the East of England–Cambridge Central Research Ethics Committee (10/H0308/50).

### Participants

Participants consisted of community-dwelling older healthy controls, and patients diagnosed with either MCI or Alzheimer's disease by secondary healthcare services (Table 1). The cognitively frail individuals were defined by underperformance on cognitive tests, without any subjective memory complaints or clinical diagnosis of dementia, MCI, or other significant neurologic and psychiatric illness. A group of cognitively frail adults was recruited from the participants who had been assessed at home as part of the Cam-CAN 3000 home assessment (Shafto et al., 2014), but who had not been recruited into the Cam-CAN 700 or Cam-CAN 280 reassessments. These healthy cognitive controls scored >25/30 on MMSE or >88/100 on the ACE-R during the home interview. The Cam-CAN home visits acquired lifestyle and cardiovascular risk characteristics (alcohol and smoking, hypertension, history of stroke and heart attack).

**Table 1.** Demographic and cognitive screening results for the four study populations^*a*^

	Controls	Cognitively Frail	MCI	Alzheimer
Group size (female)	38 (17 F)	26 (14 F)	15 (4 F)	11 (6 F)
Age (yr)	72.19 ± 8.88	79.98 ± 9.50	75.54 ± 7.60	74.53 ± 11.17
Education (yr)	14.97 ± 3.86	11.07 ± 2.88	16.68 ± 4.99	11.55 ± 3.54
Hearing left (dB)	51.92 ± 13.32	44.15 ± 16.06	52.88 ± 14.77	54.80 ± 10.01
Hearing right (dB)	53.55 ± 12.44	43.38 ± 16.35	57.63 ± 9.40	50.00 ± 12.01
MMSE (/30)	28.34 ± 1.47	26.07 ± 2.28	26.37 ± 2.73	23.20 ± 2.78
ACE-R (/100)	93.71 ± 3.04	80.92 ± 6.01	83.68 ± 8.41	68.6 ± 8.11
ACE-R memory (/26)	23.63 ± 1.94	18.38 ± 3.69	16.81 ± 6.09	10.7 ± 3.43
Training test (/100)	65.78 ± 23.55	50.96 ± 20.59	44.16 ± 24.02	37.50 ± 27.95

*^a^*MCI, Symptomatic MCI after secondary/tertiary memory clinic assessment (for comparisons between groups, see Fig. 2*B*).

In addition, patients were recruited from local specialist memory clinics who had MCI or probable Alzheimer's disease diagnosed according to Petersen and McKhann criteria, respectively (McKhann et al., 2011; Petersen et al., 2014). Most MCI/AD patients had positive CSF biomarker status for Alzheimer's disease pathology, or clinical follow-up to confirm the diagnosis. Participants were recruited from either sex, were older than 50 years, and were fluent speakers in English, with mental capacity to consent. Participants did not have any significant psychiatric illness or established neurologic condition (other than MCI or Alzheimer's disease in the patient groups).

### EEG/MEG and MRI acquisition

We used EEG/MEG to quantify neurophysiological dysfunction, as used in studies of healthy successful aging (Vlahou et al., 2014; Tsvetanov et al., 2015; Coquelet et al., 2017; Price et al., 2017), and early signatures of MCI and Alzheimer's disease (Osipova et al., 2005; Stam et al., 2006; de Haan et al., 2012; Maestú et al., 2015; Hughes et al., 2019; Kocagoncu et al., 2020). EEG/MEG data were acquired using the Elekta Vector View system with 204 planar gradiometers and 102 magnetometers. Simultaneous EEG data were acquired using a 70-channel Easycap. Participants' horizontal and vertical eye movements, and the cardiac activity were recorded using bipolar electro-oculogram and electro-cardiogram electrodes. Five head position indicator coils were placed on the EEG cap, to track the head position every 200 ms. For coregistration of the participant's T1-weighted MRI scan to the MEG sensors, three fiducial points (nasion, left, and right pre-auricular) and a minimum of 100 head shape points were digitized using Polhemus digitization.

Participants were seated in a magnetically shielded room (IMEDCO) and positioned under the MEG scanner. Auditory stimuli were delivered binaurally through MEG-compatible ER3A insert earphones (Etymotic Research). The delay in sound delivery because of the length of earphone tubes and sound card was 26 ± 2 ms on average. Visual stimuli were presented on the screen positioned 1.22 m in front of the participant's visual field. Simultaneous EEG/MEG was recorded continuously at 1000 Hz with a high-pass filter of 0.03 Hz. Before the EEG/MEG recording, participants performed an automated hearing test in the MEG scanner, to make sure that the earphones were working properly. They were presented pure tones at the frequency of 1000 Hz to either ear with varying loudness. Participants were instructed to press the button when they heard the tone. The mean hearing levels of each group are given in Table 1, where the normal range is expected to fall within 45-75 dB.

T1-weighted structural images were acquired on a Siemens 3T Magnetom Prisma MRI Scanner using an MPRAGE sequence (TR = 2250 ms, TE = 2.99 ms; inversion time = 900 ms; flip angle = 9 degrees; FOV = 256 mm × 240 mm × 192 mm; voxel size = 1 mm isotropic; GRAPPA acceleration factor = 2; acquisition time = 4 min 32 s). Four participants did not tolerate MRI because of claustrophobia.

### Stimuli

The stimuli consisted of abstract images and pure tones. There were four images with distinct patterns. The tones had the following frequencies: 503, 719, 1021, and 1451 Hz. Harmonic tones were avoided by choosing frequencies of prime numbers and varying them by at least three semi-tones. There were four types of trials. (1) Standard (STD) trials were image-tone pairs that participants trained on before the task. STD pairs were the trials presented most frequently. (2) DA trials presented the same images of the STD pairs but by shuffling the sounds. The DA trials were expected to capture the binding effect arising from a mismatch in association. (3) DN trials presented the STD images with rare deviant tones. The frequencies used for the DNs were 599, 857, 1017, and 1733 Hz. The DN trials were expected to capture the novelty effect, and were essentially the deviants used in conventional mismatch paradigms. The deviant trials were expected to induce a mismatch response with respect to the response to STD trials. (4) Target trials: The STD pairs, where the image was bound by a red circle. Target trials were included to make sure participants were attending to the stimuli. There were in total 1000 STD trials; and DA, DN, and target trials were presented 48 times each. Therefore, the DA, DN, and the targets were each encountered 4% of the time each, whereas STDs 88% of the time.

### Paradigm

The cross-modal oddball paradigm depends on both change detection and associative binding. This has two advantages. First, as MEG recording has lower signal-to-noise ratio in the subcortical areas and deeper sources compared with signal coming from superficial cortices (Goldenholz et al., 2009), the task was specifically designed to capture the indirect response in the superior temporal gyri (STG) and inferior frontal gyri (IFG), which are dependent on hippocampal associative learning. Second, the integrity of the auditory and frontal cortex is preserved until late stages of Alzheimer's disease, allowing us to control for atrophy of the cortical generators of the mismatch response. The task was easy to perform both by all participant groups and required minimal training (reducing potential confounds, e.g., education and cognitive strategies on performance).

**Figure 1.**
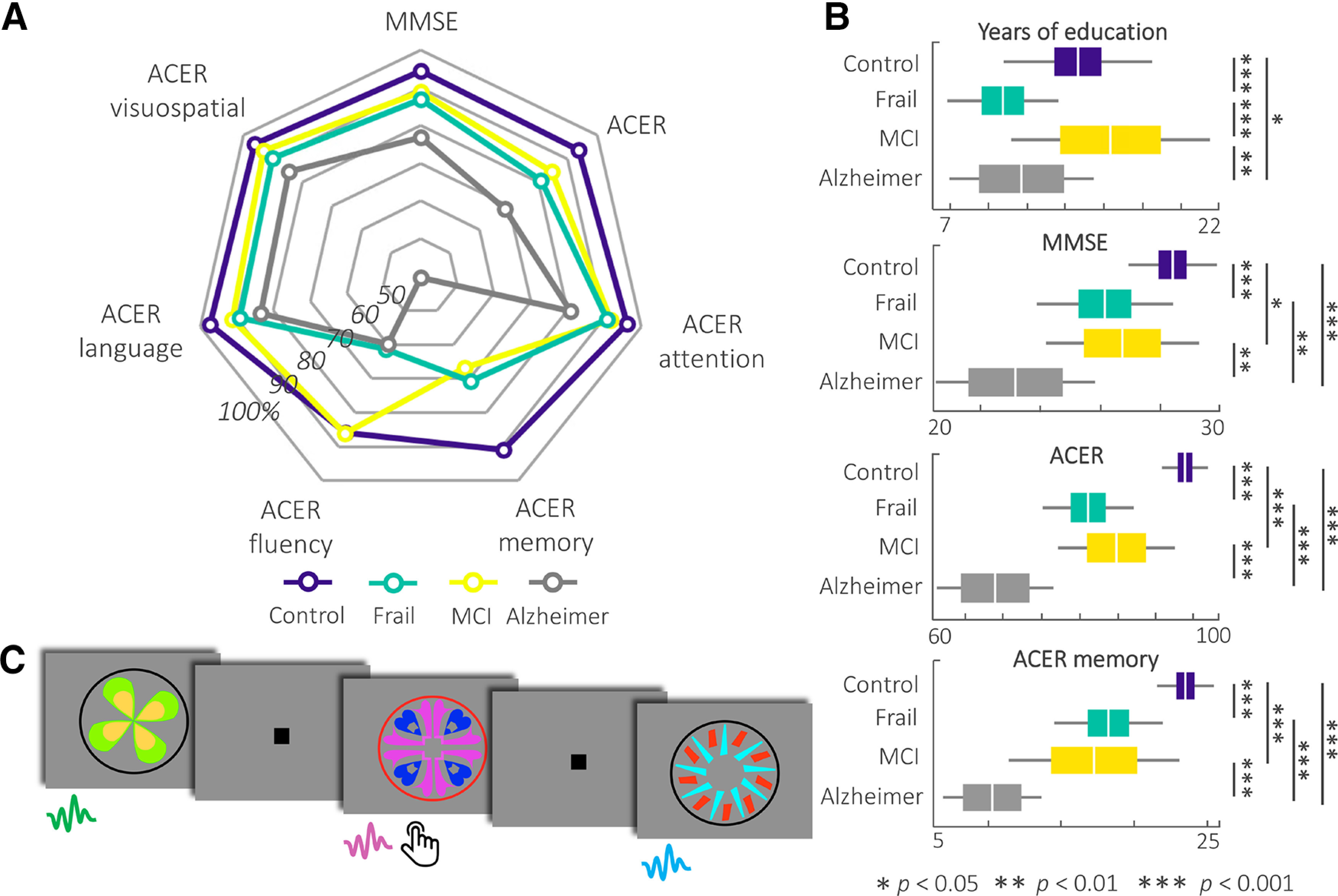
***A***, Radar chart represents the group means for the neuropsychological tests converted to percentages against the maximum score in each test for ease of comparison across groups. The performance of the cognitively frail group overlaps with the MCI across all tests except for ACE-R fluency. ***B***, Group differences in education levels and neuropsychological tests. The cognitively frail group had lower education levels than the controls. On the neuropsychological tests, the cognitively frail performed similar to the MCI group. ***C***, Example stimuli from the cross-modal oddball task. The images were presented together with paired sounds after the 300 ms lag. Participants were asked to press the button whenever they saw a red circle around the image. MCI, Symptomatic MCI after secondary/tertiary memory clinic assessment.

Images were presented centrally on a gray screen bounded by a black circle for 800 ms. Then, 300 ms after image onset, the tone was played for 500 ms (see Fig. 1*A*). The 300 ms lag was introduced to allow participants to form predictions about the upcoming auditory stimuli. In between trials, a black fixation square was presented for a jittered period of 300-500 ms, resulting in a stimulus onset asynchrony between 1000 and 1200 ms. E-Prime 2 (Psychology Software Tools) was used to present the stimuli and send triggers to the scanner.

In the training phase, participants were presented in total four images and four tones (i.e., STD pairs), 25 times each, and were instructed to try to remember the pairings between the images and the tones. After the training, participants performed a short test where they listened to the four tones twice in a randomized order. After each tone, they were shown four images (i.e., chance level of 25%) on the screen and were asked to select the image that was paired with that tone. Irrespective of the participant's performance, training was repeated only once. Following the training, participants moved on to the main task. Trials were presented in a different randomized order for each participant across four 5-min-long blocks. Participants were instructed to pay attention to the images and press the button with their right index finger when the image was bound by a red circle.

### EEG/MEG preprocessing and source localization

The raw EEG/MEG data were preprocessed using MaxFilter 2.2.12 (Elekta Oy). MaxFiltering included detection and interpolation of bad sensors, signal space separation to remove external noise from the data, and head movement correction. Cardiac and blink artifacts were detected and removed using an independent component analysis with 800 maximum steps and 64 principal components via the EEGLAB toolbox (Delorme and Makeig, 2004). The independent component time series were correlated with EOG and ECG time series and spatial templates. The components that revealed higher than *z* = 3 in the temporal and *z* = 2 in the spatial dimension were removed, and the remaining time series of the independent components were reconstructed. On average, 2.38 blink components (SD = 0.58) and 1.30 cardiac components (SD = 0.49) were removed.

Data were further processed in SPM12 (www.fil.ion.ucl.ac.uk/spm). Data were bandpass filtered between 0 and 40 Hz using a fifth-order Butterworth filter. The continuous data were epoched between −100 and 500 ms from the sound onset. OSL's artifact rejection algorithm (www.github.com/OHBA-analysis/osl-core) was used to remove remaining artifacts (e.g., motor). Bad channels and trials marked by the algorithm were removed. On average, 53.04 (4.6%; SD = 35.88) trials and 10.76 channels (2.8%; SD = 5.85) were removed per participant. Trials were averaged within condition, using robust averaging. Low-pass filter was reapplied to correct for the high-frequency noise introduced by robust averaging.

The EEG/MEG data were source localized using all sensor data: magnetometers, gradiometers, and EEG (Henson et al., 2009). The source space was modeled with a medium-sized cortical mesh consisting of 8196 vertices via inverse normalization of SPM's canonical meshes. Sensor positions were coregistered to the native T1-weighted MPRAGE scans using the fiducial and head shape points after removing digitization points around the nose. SPM's canonical template brain was used for participants who did not tolerate the MRI scan. Single shell and Boundary Element models were used for forward modeling of MEG and EEG data, respectively. Evoked signal was estimated over the trials using the COH solution in SPM, which imposes spatial smoothness on the prior covariance matrix. All inversion accuracies were >80%, as measured by the proportion of variance explained in the sensor data (mean = 93.62; SD = 3.63).

**Figure 2.**
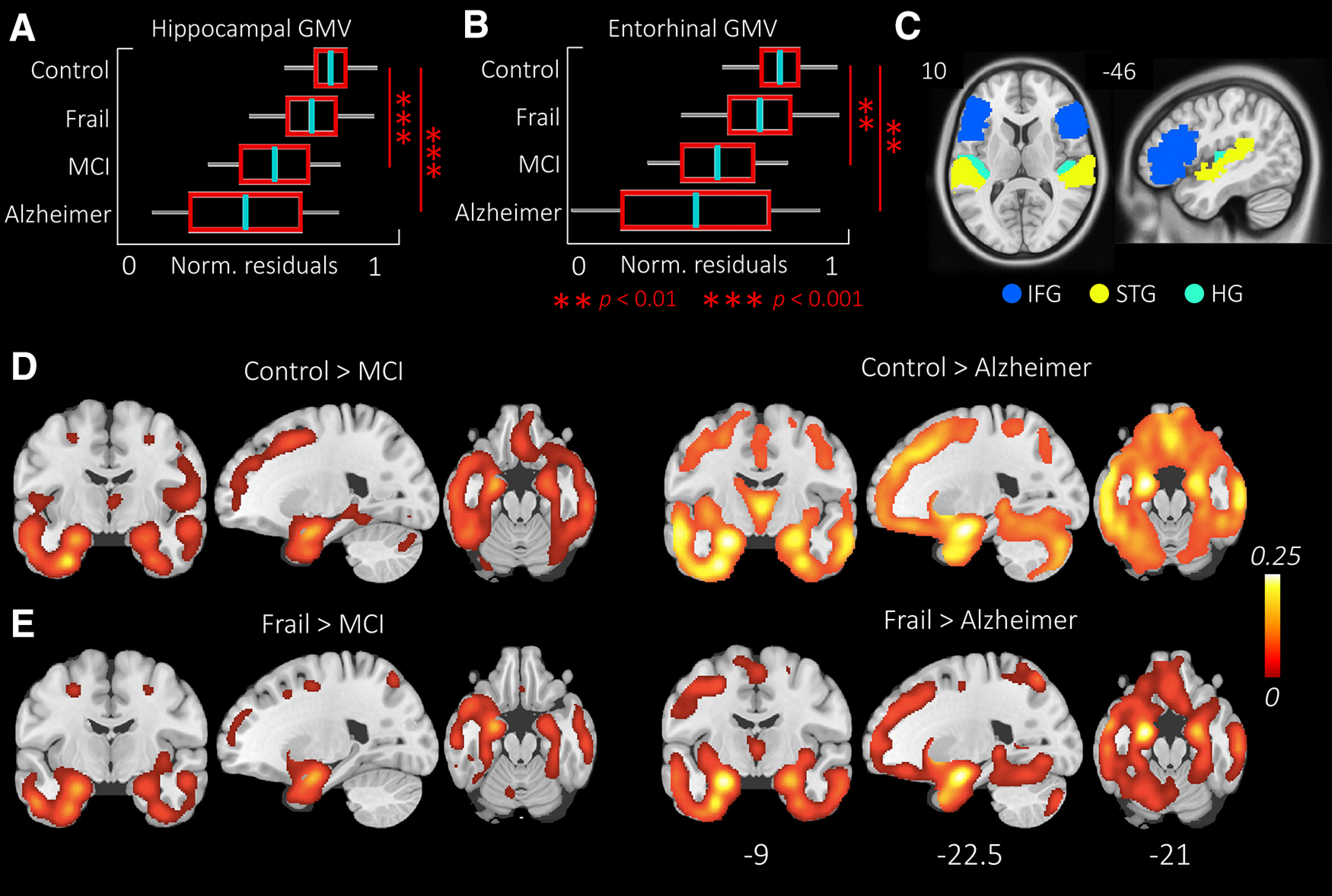
***A***, ***B***, GMV differences across groups in the hippocampus and entorhinal cortex. Boxplots represent the normalized residuals after correcting for differences in age and TIV. There were no significant differences in volume between the cognitively frail and the control group. ***C***, Six ROIs used in the RMS analysis. ROIs comprise IFG, STG, and HG bilaterally. ***D***, ***E***, The contrast images from the voxel-based morphometry analysis. Control and the cognitively frail show similar patterns of GMV compared with the MCI and Alzheimer's disease groups. For the GMV analysis of the lateral frontotemporal ROIs used in the RMS analysis, see Extended Data Figure 2-1. MCI, Symptomatic MCI after secondary/tertiary memory clinic assessment.

Although neurophysiological responses in the hippocampus are difficult to detect with EEG/MEG, owing to its depth, a strong mismatch response can be recorded from temporal cortex, where sensory predictions are assumed to be established from hippocampal-dependent cross-modal associative learning. We therefore focus on the mismatch response in the lateral temporal auditory and frontal cortex, as activated in conventional auditory oddball paradigms (Pekkonen, 2000; Garrido et al., 2009; Hughes and Rowe, 2013; Phillips et al., 2016; Hughes et al., 2018). The source localized data were extracted from six areas taken from the Automated Anatomical Labeling atlas: Heschl's gyrus (HG), STG, and IFG bilaterally (see Fig. 2*C*). The ROI masks were resliced to 1 mm isotropic thickness to allow maximum data extraction. For each participant and condition, the data were extracted from the peak within all the vertices that constitute each ROI. This is to maximize the signal-to-noise ratio in the data, and to account for individual variability in source activity. We had two contrasts of interest in the analyses: the STD-DN contrast captures the novelty mismatch effect, whereas the STD-DA contrast captures the associative mismatch effect.

### MRI preprocessing and gray matter analysis

The T1 image was rigid-body coregistered to an MNI template and then corrected for image inhomogeneity and segmented into six tissue classes (gray matter, white matter, CSF, bone, soft tissue, and residual noise) using SPM's unified segmentation algorithm (Ashburner and Friston, 2005). The native space gray and white matter images for all participants were then submitted to diffeomorphic registration (DARTEL) (Ashburner, 2007) to create group template images. The group template was then normalized to the MNI template via an affine transformation, and the combined normalization parameters (native to group template and group template to MNI template) were applied to each individual participant's gray matter image, including modulation to preserve local volume. ROIs from the Harvard-Oxford atlas were then used to extract mean regional GMV from the bilateral hippocampal and entorhinal ROIs for each participant. The GMVs were compared across groups using ANCOVAs where age and total intracranial volume (TIV) were set as covariates.

To calculate local gray matter atrophy at the whole-brain level, we used voxel-based morphometry. Gray matter segments were thresholded with an absolute masking level of 0.1, and were smoothed with a Gaussian kernel at 8 mm FWHM. Gray matter volumes (GMVs) were compared across groups in pairwise *t* contrasts in GLMs accounting for differences in age and TIV. The cluster level *p* values were corrected for multiple comparisons using the familywise error after a cluster defining threshold of *p* < 0.05.

### Root mean square (RMS) and statistical analyses

To investigate differences in time series, the RMS of the time series at each ROI and trial were smoothed using a moving average at every 50 time points, to remove jumps. The RMS at each time point was then modeled using GLMs accounting for differences in age and hearing levels, and tested for within-group task effects by using *t* contrasts. The contrasts compared the signal intensity between the DA-STD and DN-STD. The tests comparing the deviant effects were performed first within each participant group, to reveal task-specific effects. Second, these differences were tested across groups to test for interaction effects between conditions and groups. The observed cluster masses in the GLMs were corrected for multiple comparisons using permutation cluster statistics, by bootstrapping the design matrix using 1000 permutations at *p* = 0.05. The mean of the time series within each contrast was calculated for each participant within the 200-500 ms time window after removing outliers. This time window was selected because task effects were strongest after the N100. The linear relationship between these metrics and predictor variables was further tested through GLMs across the sample, including age as a covariate, and after removing outliers. The predictors of interest were years of education, ACE-R total and memory subscale scores, and hippocampal and entorhinal GMVs.

## Results

### Sample characteristics

Sample characteristics and scores on neuropsychological tests were compared across the groups using ANOVAs. Age (*F*_(3,87)_ = 3.82; *p* = 0.012) and years of education (*F*_(3,87)_ = 11.34; *p* < 0.001) differed between groups. Tukey's HSD tests showed that the cognitively frail group was older than controls (*p* = 0.006). The duration of formal education was longer in those in the control group than the cognitively frail (*p* < 0.001), and Alzheimer's disease group (*p* = 0.032). The MCI group had had longer education than the cognitively frail (*p* < 0.001) and Alzheimer's disease (*p* = 0.001) groups. Hearing levels were tested for group differences using both ANOVA and ANCOVA (to control for differences in age). There were no significant differences in hearing in the left ear. In the right ear, there was a group difference (*F*_(3,86)_ = 4.70; *p* = 0.004): the hearing of the cognitively frail was lower than control (*p* = 0.017) and MCI (*p* = 0.005) groups. When adjusting for the differences in age (*F*_(3,85)_ = 3.02; *p* = 0.034), the hearing levels on the right were still lower in the cognitively frail group compared with the MCI group (*p* = 0.019). χ^2^ tests compared the prevalence of lifestyle and cardiovascular risk factors between control and the cognitively frail groups. The prevalence of daily alcohol consumption was lower in the cognitively frail group (19%) compared with controls (43%) (χ^2^_(1)_ = 6.11; *p* = 0.006). The prevalence of hypertension was higher in the cognitively frail group (52%) compared with the controls (35%) (χ^2^_(1)_ = 2.74; *p* = 0.048). There were no significant differences between groups in the prevalence of smoking, history of stroke, or heart attack.

### Cognitive results

Cognitive scores were tested for group differences after controlling for differences in age (Table 1). The MMSE (*F*_(3,86)_ = 17.64; *p* < 0.001), ACE-R total score (*F*_(3,86)_ = 55.41; *p* < 0.001), ACE-R's subscales in memory (*F*_(3,86)_ = 37.35; *p* < 0.001), attention (*F*_(3,86)_ = 9.05; *p* < 0.001), fluency (*F*_(3,86)_ = 13.87; *p* < 0.001), language (*F*_(3,86)_ = 7.90; *p* < 0.001), and visuospatial skills (*F*_(3,86)_ = 11.15; *p* < 0.001) showed strong differences across the groups (Fig. 1*A*). Results of the pairwise *post hoc* comparisons are given in Figure 1*B*. The cognitively frail group performed similarly to the MCI group across all cognitive tests, except for the fluency subscale, where their scores were significantly lower than the MCI group (*p* < 0.001). All four groups performed above chance level on the training test. The scores were significantly different across groups (*F*_(3,86)_ = 5.60; *p* = 0.001). *Post hoc* comparisons showed that the controls performed significantly better than the MCI (*p* = 0.015) and Alzheimer's disease (*p* = 0.006) groups. There were no significant differences between the training scores of the cognitively frail group and other groups.

### Gray matter atrophy

Mean hippocampal GMV, entorhinal GMV, total GMV, and TIV were compared across the groups, corrected for age and TIV using ANCOVA. There were no significant differences between groups for TIV or total GMV. However, hippocampal (*F*_(3,86)_ = 10.35; *p* < 0.001) and entorhinal (*F*_(3,86)_ = 7.62; *p* < 0.001) GMVs showed a main group effect (Fig. 2*A*,*B*). The hippocampal GMV in the control group was significantly larger compared with the MCI (*p* < 0.001) and Alzheimer's disease groups (*p* < 0.001). Similarly, the entorhinal GMV of the control group was larger compared with the MCI (*p* = 0.001) and Alzheimer's disease groups (*p* = 0.003). The hippocampal and entorhinal volumes of the cognitively frail group were similar to the control group.

10.1523/JNEUROSCI.0697-21.2021.f2-1 Figure 2-1 Gray matter volume analysis of the lateral frontotemporal network. In Alzheimer's disease. atrophy of the auditory cortex comes at the later isocortical Braak stages (i.e., V-VI) along with other neocortical areas such as the frontal cortex. Therefore, in the early stages of Alzheimer's disease, the integrity of the auditory cortex is not expected to be compromised, and consequently, diminished deviant responses observed in our clinical population are not expected be attributed to the atrophied auditory cortex. To provide support for this claim, we performed the confirmatory GMV comparisons across the four groups within the lateral frontotemporal ROIs which were used to extract neurophysiological signals from. We performed ANCOVAs to test for group differences while accounting for differences in age and TIV. As expected, there were no significant effect of the group in any of the six ROIs, confirming that the differences we find in the deviant responses could not be attributed to local GM atrophy. C, Controls; F, cognitively frail; AD, Alzheimer's disease. Download Figure 2-1, TIF file.

Atrophy was tested at the voxel level, using voxel-based morphometry (Table 2). As expected, the control group had significantly higher GMV in bilateral temporal cortices and hippocampi compared with the MCI and AD. We found a similar pattern comparing cognitively frail group to MCI and Alzheimer's disease group, although cluster extents were smaller (Fig. 2*D*,*E*). In order to confirm that the differences we observe in the neurophysiological responses could be because of local GM atrophy of the lateral frontotemporal areas, we tested the GMV of the lateral frontotemporal areas bilaterally (i.e., IFG, STG, HG), and report that the GMVs of the superficial areas do not differ across groups (Extended Data Fig. 2-1).

**Table 2.** Voxel-based morphometry of volume differences between groups^*a*^

Test	Cluster peak	*x*, *y*, *z* (mm)	Cluster extent	*k*	*p*_FWE_
Control>MCI	R middle temporal	58, −39, 3	R superior temporal, R inferior temporal, R hippocampus, R parahippocampal, R fusiform	26,826	<0.001
	L middle temporal	−52, −54, 12	L fusiform, L inferior, L hippocampus, L parahippocampal	24,165	<0.001
	L middle frontal	−21, 41, 30	L superior frontal	2063	0.004
	L precentral	−40, 6, 36	L inferior frontal	1407	0.020
Control>Alzheimer	R hippocampus	28, −13, −11	L/R parahippocampal, L fusiform, L/R putamen, L caudate, L superior frontal, L hippocampus, L insula, L medial frontal	55,790	<0.001
	L middle temporal	−57, −16, −9	L inferior temporal, L middle temporal	7744	<0.001
	R middle temporal	64, −12, −20	R inferior temporal	4539	<0.001
	L postcentral	−50, −18, 34	L inferior parietal	1258	0.028
Cognitively frail>MCI	L inferior temporal	−34, 5, −35	L hippocampus, L parahippocampal, L middle temporal, L fusiform	7546	<0.001
	R inferior temporal	54, −19, −17	R middle temporal, R hippocampus, R fusiform	2070	0.004
Cognitively frail>Alzheimer	L hippocampus	−22, −13, −14	L parahippocampal, L fusiform, L inferior temporal	3723	<0.001

*^a^*Columns in the table indicate the peak cluster, coordinates of the peak in millimeters, the extent of the cluster, the cluster mass, and corrected *p* value for the cluster, respectively. *k*, Cluster mass; p_FWE_, *p* value corrected for familywise error rate; MCI, symptomatic MCI after secondary/tertiary memory clinic assessment.

### Cross-modal mismatch responses

**Figure 3.**
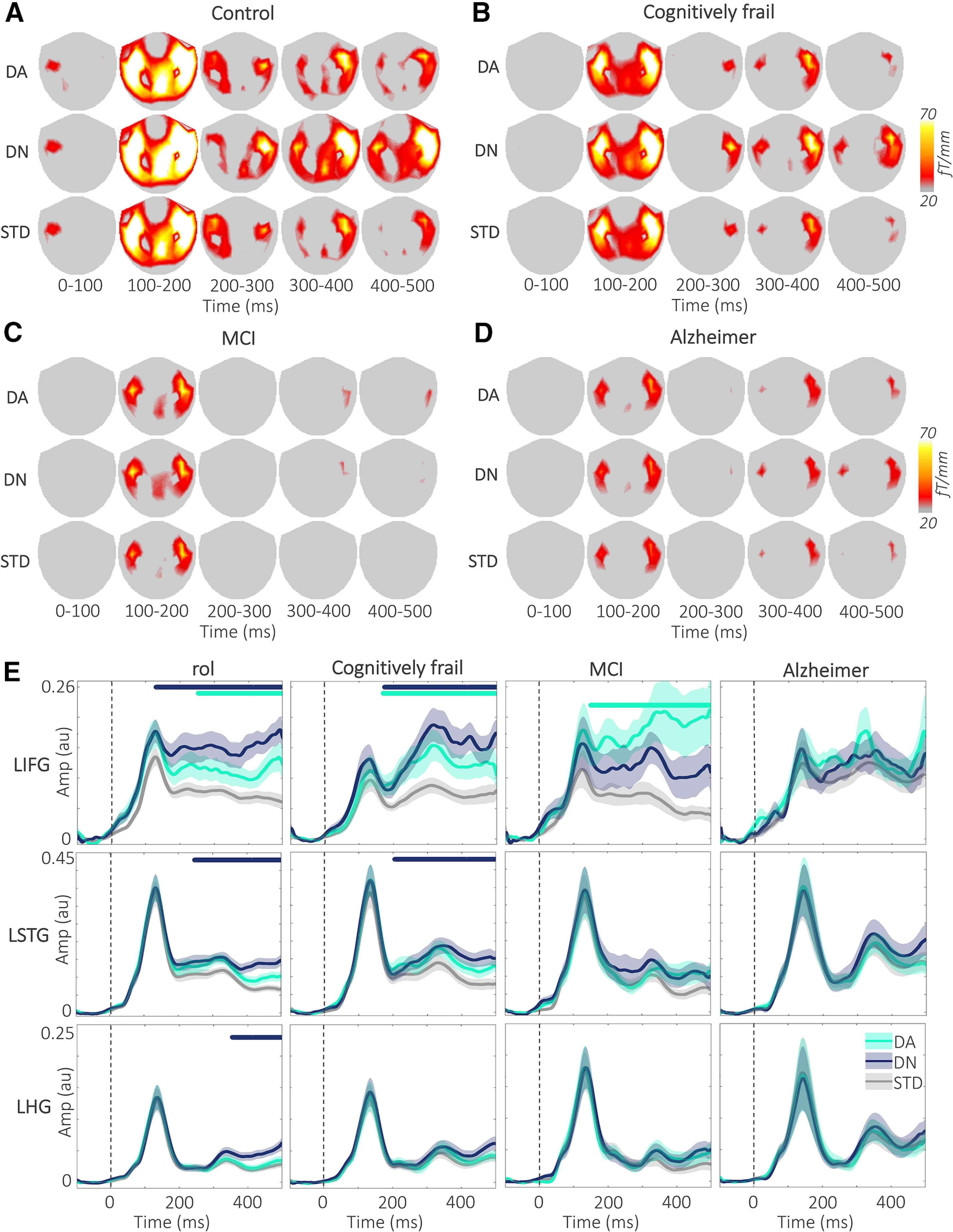
DA and DN responses by group. ***A****-****D***, Topoplots represent the mean gradiometer activity across the scalp for the DA, DN, and STD conditions in 100 ms time windows in four groups. The DN amplitude is higher after the N100 peak, compared with both DA and STD conditions in control and cognitively frail groups. The gradiometer activity in the MCI and Alzheimer's disease groups is weaker compared with the control and cognitively frail groups. ***E***, Plots represent the RMS time series for the left hemisphere ROIs for simplicity. Dashed vertical lines indicate the sound onset. The amplitude differences between the deviants and the STD in the frontal regions were larger than the temporal regions, and the deviant effects are stronger in the control and cognitively frail groups; and there is considerably higher variance in the MCI group. For the renderings of the source activity, see Extended Data Figure 3-1. Amp, Amplitude; AU, arbitrary units; fT, femtotesla; LHG, left HG; LIFG, left IFG; LSTG, left STG.

Figure 3*A-D* displays the gradiometer topoplots for each condition in 100 ms time windows across the groups. Following N100, topoplots represent a strong burst of bilateral activity in frontal and temporal sensors that is sustained until the end of the epoch. Compared with the DA and STD, DN induced a stronger and more widespread activity across the frontotemporal sensors. The gradiometer topoplots are given here for visualization only; statistical comparisons were made in the *a priori* source space ROIs.

10.1523/JNEUROSCI.0697-21.2021.f3-1 Figure 3-1 Source activity: The renderings display mean source activity in 100 ms moving time windows in the Control group. In line with the topoplot activity and our RMS findings, here we find the activation of a bilateral frontotemporal network peaking at 100 ms. Further, compared with the STD condition, deviant conditions show stronger and more widespread activity in the frontotemporal regions 200 ms after the sound onset. Download Figure 3-1, TIF file.

We tested the time series of each deviant with respect to the STD, within the 6 ROIs (Table 3; Fig. 3*E*). We found strongest effects for the DN in the bilateral IFG early in the epoch, following the onset of the sound. The effects seen in the cognitively frail group mirrored the controls. Further, DN effects were found across all the ROIs in the control group. MCI and Alzheimer's disease groups showed no significant novelty effects in the IFG, and weaker clusters limited to STG and HG. DA effects were found in the IFG across all groups, and in overlapping time windows starting at ∼200 ms after the sound onset.

**Table 3.** Regional differences in the response to DN and DA compared with STD trials*^a^*

Contrast	ROI	Group	*k*	*p_cor_*	Time (ms)
STD-DN	LIFG	Control	−1478.03	<0.001	31-500
		Frail	−938.79	<0.001	175-500
	RIFG	Control	−1631.81	<0.001	1-500
	LSTG	Control	−715.93	0.002	255-500
		Frail	−449.99	0.021	331-500
	RSTG	Control	−1068.44	0.003	172-500
		Frail	−870.95	0.003	197-500
		Alzheimer	−279.53	0.041	391-500
	LHG	Controls	−348.23	0.034	355-500
	RHG	Controls	−464.34	0.021	305-500
		Frail	−253.10	0.046	380-500
		MCI	−261.62	0.041	350-470
STD-DA	LIFG	Control	−480.41	0.023	254-500
		Frail	−680.74	0.014	171-497
		MCI	−974.42	0.004	150-500
	RIFG	Control	−944.45	<0.001	192-500
		Frail	−561.03	0.013	188-458
		Alzheimer	−929.57	0.003	162-500

*^a^k*, Cluster mass; LHG, left HG; LIFG, left IFG; LSTG, left STG; p_cor_, corrected *p* value; RHG, right HG; RIFG, right IFG; RSTG, right STG. For the log-transformed version of this analysis, see Extended Data Table 3-1.

10.1523/JNEUROSCI.0697-21.2021.tab3-1 Extended Data Table 3-1 Regional differences in the response to novelty deviants (DN) and associative deviants (DA) compared to standard trials. In this version of the analysis, we performed the statistical comparisons on the log transformed squared RMS time series, to ensure the normality assumption of the GLMs is met. The results of the RMS and log transformed RMS time series largely overlap. However in the latter analysis task-group interaction effects do not reach statistical significance. *k: Cluster mass; p_cor_: Cluster corrected p-value*. Download Table 3-1, DOCX file.

### Clinical and structural correlates of the cross-modal mismatch

**Figure 4.**
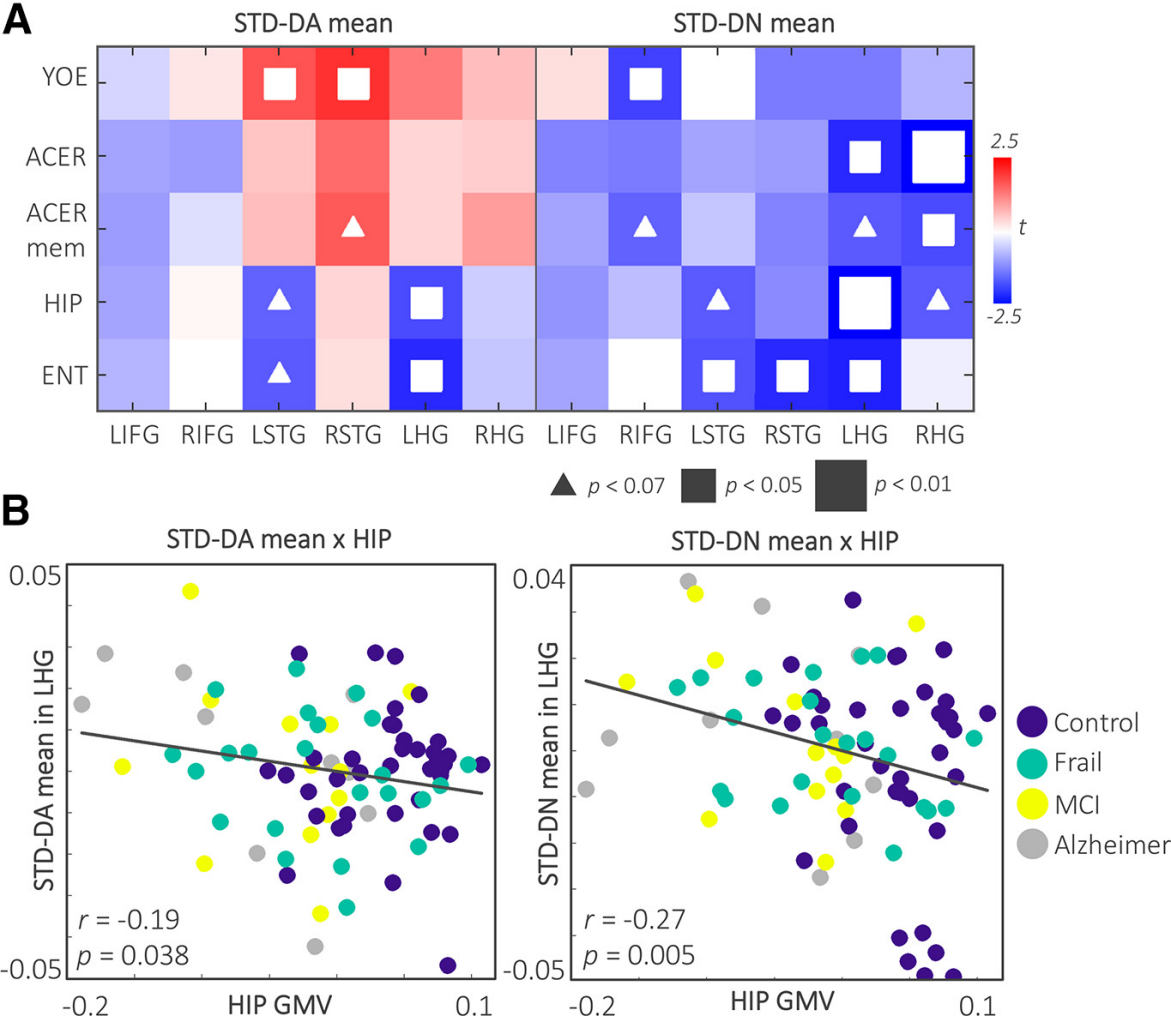
Neurophysiological responses are related to clinical and volumetric differences between individuals. ***A***, The t-map displays the GLM results across predictors and DA and DN mean responses for each ROI. White squares represent significant effects. The effects are stronger for the LHG across the ROIs, and for the DN compared with the DA. ***B***, Scatterplots represent the negative relationship between the DA and DN means in the LHG with the hippocampal GMV across the sample. ACE-R mem, ACE-R memory subscale; ENT, entorhinal GMV; HIP, hippocampal GMV; LHG, left HG; LIFG, left IFG; LSTG, left STG; RHG, right HG; RSTG, right STG; RIFG, right IFG; YOE, years of education.

To assess how the deviant responses relate to clinical severity, education, and medial temporal lobe atrophy, the linear relationships between the EEG/MEG contrast means at 200-500 ms and each predictor variable were tested using GLMs (Fig. 4*A*) while controlling for differences in age. This revealed strong relationships between the DN mean in the left HG and right HG with ACE-R total and ACE-R-memory subscale scores: the higher the scores on cognitive tests, the more negative (toward normal) the DN was. A strong negative relationship between the hippocampal and entorhinal volumes and the deviant response was observed for the left hemisphere ROIs, particularly the left HG. This suggests that medial temporal atrophy is associated with a reduced deviant response, although the mismatch negativity response arises from extrahippocampal auditory cortex. This negative relationship was stronger for the DN compared with the DA. Education showed moderate positive relationships with the DA in the left STG and right STG, whereas it showed a negative relationship with the DN mean in right IFG.

We calculated the partial correlations among predictor variables correcting for differences in age. Education showed positive correlations with ACE-R total (*r* = 0.40; *p* < 0.001) and ACE-R memory subscale scores (*r* = 0.35; *p* = 0.001), but did not correlate with hippocampal and entorhinal volumes. ACE-R total score correlated with both hippocampal (*r* = 0.53; *p* < 0.001) and entorhinal GMV (*r* = 0.40; *p* < 0.001). Similarly, ACE-R memory subscale score positively correlated with hippocampal (*r* = 0.55; *p* < 0.001) and entorhinal GMV (*r* = 0.41; *p* < 0.001).

## Discussion

The principal result of this study is that community-dwelling cognitively frail individuals do not resemble people with MCI or Alzheimer's disease, in terms of their structural or neurophysiological profile, despite similar levels of underperformance on cognitive screening tests. The poor cognitive performance of the cognitively frail participants should not simply be interpreted arising from latent Alzheimer pathology or undiagnosed amnestic MCI. Population screening using standard cognitive tests (e.g., MMSE, or ACE-R) is therefore unlikely to selectively identify those with latent Alzheimer's disease pathology without additional biomarker evidence of pathology. There are other associations of cognitive impairment, including lower educational level, hearing impairment, and cardiovascular risk factors. Both structural and neurophysiological features of the cognitively frail group were similar to controls. Structural analyses revealed a higher GMV in the latero-medial temporal cortices bilaterally in the control and cognitively frail compared with MCI and Alzheimer's disease groups. Like the controls, the cognitively frail showed stronger DA and DN responses compared with MCI or Alzheimer's disease in relation to hippocampal and entorhinal volumes.

The cross-modal oddball task was designed to induce deviant responses from the superficial frontotemporal cortex, as neurophysiological markers of hippocampal-dependent associative learning. Alzheimer's patients show impairments in both sensory and associative memory, reduced medial temporal lobe activity to novelty (Sperling et al., 2003; Dickerson et al., 2005), and reduced electrophysiological response to oddballs (Engeland et al., 2002; Lee et al., 2013; Ruzzoli et al., 2016). We confirmed that Alzheimer's patients show reduced DA and DN responses. Neurophysiological profiles of the control and cognitively frail overlapped, and were significantly stronger compared with MCI and Alzheimer's disease groups. Task effects of the DN responses were observed across all ROIs for the control and cognitively frail. The group-task interaction effects showing stronger DA and DN responses for the controls and cognitively frail were located in right Heschl's, bilateral superior temporal, and IFG. In other words, the neuropsychologically impaired cognitively frail group does not show the neurophysiological signatures of early Alzheimer's disease. From a theoretical perspective, it is interesting to note that the response associated with DAs was weaker than DNs, and did not differ qualitatively in terms of timing or distribution across our ROIs. We had expected the DA response to be more hippocampal-dependent, and hence more impaired with Alzheimer's pathology, but our current analyses suggest it was qualitatively similar to the more typical DN response.

To explore the differences between the community-based cognitively frail and Alzheimer's disease or MCI, we tested volumetric differences in medial temporal lobe. Structurally, early Alzheimer's disease is characterized by atrophy in the medial temporal lobe as a function of tau burden (Braak et al., 2006; Schwarz et al., 2016). Recent studies of cognitive frailty have suggested frontotemporal and subcortical atrophy (Del Brutto et al., 2017; Gallucci et al., 2018), increased white matter hyperintensities (Avila-Funes et al., 2017; Del Brutto et al., 2017; Sugimoto et al., 2019), and decreased white matter microstructure integrity (Avila-Funes et al., 2017). We did not find structural differences between the control and cognitively frail in medial temporal lobe structures. The community-based groups showed significantly larger hippocampus and entorhinal volumes compared with patients with MCI and Alzheimer's disease, and did not show early structural signatures of Alzheimer's disease. The difference between our study and the previous work may lie in the epidemiological approach to baseline recruitment through the Cam-CAN 3000 cohort, rather than clinical referral pathways.

The neuropsychological profile of the cognitively frail resembled MCI group. They scored lower than the healthy controls on every ACE-R subscale. Compared with the MCI patients, they were more impaired on fluency, which might indicate an underlying executive deficit. Previous studies have suggested that the neuropsychological profile of cognitive frailty differs from MCI in episodic memory with domains of language, visuospatial skills, and executive function relatively spared (Collie and Maruff, 2000). The cognitive impairment profile in frail adults has been described in terms of deficits in executive function and attention. Frail adults tend to not use cues effectively to retrieve stored information (Canevelli et al., 2015; Delrieu et al., 2016), have slower reaction times (O'Halloran et al., 2014; Robertson et al., 2014), show lower meta-cognitive awareness, and show error monitoring (Amanzio et al., 2017).

However, many previous studies have focused on cognitive impairment in the context of physical frailty, rather than defining cognitive frailty in terms of poor cognitive function in a nonclinical community-dwelling cohort. The cognitive underperformance of our cognitively frail might partly be attributed to their shorter education, coupled with a bias in most cognitive tests toward the better educated (Huppert et al., 2005). That is, highly educated individuals perform better on cognitive tests, such as MMSE and ACE-R, unless scores are normalized by education (Crane et al., 2006; Mathuranath et al., 2007; Amaral-Carvalho and Caramelli, 2012).

Our findings support the hypothesis that cognitive frailty represents part of the spectrum of normal neurocognitive function, rather than incipient Alzheimer's disease. This conclusion calls for a reevaluation of the prior findings that associate cognitive frailty with higher incidence of dementia and faster cognitive decline (Buchman et al., 2007; Kojima et al., 2016; Shimada et al., 2018). These former studies have quantified the dementia incidence, including all subtypes of dementia; however, this association was highest in non-Alzheimer's dementias, particularly for vascular dementia (Panza et al., 2006; Avila-Funes et al., 2012; Gray et al., 2013; Aguilar-Navarro et al., 2016; Solfrizzi et al., 2017a). Although the link between cognitive frailty and Alzheimer's disease in previous studies is not conclusive, the two entities might share common risk factors, such as cardiovascular disease (Panza et al., 2006; Frisoli et al., 2015; Fuhrmann et al., 2019) and hearing impairment (Valentijn et al., 2005; Panza et al., 2015).

In addition to the cardiovascular risk factors (Newman et al., 2001; Patrick et al., 2002; Fuhrmann et al., 2019), the cognitive underperformance of our cognitively frail group could be a result of cumulative effects of multiple psychosocial and medical risk factors. Malnutrition (Mulero et al., 2011; Chye et al., 2018; Rietman et al., 2018), social isolation (Robertson et al., 2013), sedentary lifestyle (Landi et al., 2010), lack of intellectual cognitive activities (Jung et al., 2010), psychiatric illnesses and long-term use of antidepressants (Paulson and Lichtenberg, 2013; Gray et al., 2015), chronic inflammation (Weaver et al., 2002; Solfrizzi et al., 2017b), and lower education levels (Rogers et al., 2017) are known risk factors affecting healthy aging. Here, the cognitively frail group had significantly lower education levels compared with the controls and MCI. This is a common pattern observed in other frailty studies (Brigola et al., 2019; Margioti et al., 2020). The cognitively frail population have significantly lower occurrence of third-level education (Robertson et al., 2014), and are twice as likely to have no educational qualifications (Rogers et al., 2017). Further, strong association between educational level and frailty was linked to mediating socioeconomic, behavioral, and psychosocial factors, such as low income, chronic diseases, obesity, depression, unhealthy lifestyle, and chronic stress (Hoogendijk et al., 2014). This is consistent with the cognitive reserve hypothesis that an individual's prior education and cognitive abilities modify the resilience of brain structure to disease and injury (Stern, 2002). Longer education in early life and continuing diverse cognitive leisure activities in midlife and old age contribute to an individual's cognitive reserve, and is related to better cognitive functioning in old age (Singh-Manoux et al., 2011; Borgeest et al., 2018; Lavrencic et al., 2018; Brigola et al., 2019) and having fewer symptoms of cognitive decline and neuropathology (Mortimer et al., 2003; Chapko et al., 2018).

The study has several limitations. Because of the cross-sectional design of the study, we are unable to quantify the rates of progression or conversion to dementia from cognitive frailty. Longitudinal cognitive and neuroimaging studies would be useful to confirm the rate of conversion to Alzheimer's disease or other dementia, and potential mediators of conversion. Further, this study did not incorporate Alzheimer's disease biomarkers and instead used clinical criteria and neuropsychological criteria to define the groups. The cognitively frail group was defined using a standard threshold on ACE-R and MMSE. Future studies investigating the link between cognitive frailty and Alzheimer's disease may test for biomarkers of Alzheimer's, such as tau and amyloid-β measures acquired from blood, CSF, or positron emission tomography. Future studies may also assess the polygenic risk for Alzheimer's disease using common (e.g., APOE) and rare variants associated with the disease, which would help disentangle environmental and psychosocial risk factors from genetic risk factors contributing to cognitive frailty's etiology. Further work is needed to clarify genetic and pathology-based features of cognitive frailty in relation to Alzheimer's disease and other dementias.

Our findings provide new evidence that community-dwelling cognitively frail older adults are neurophysiologically and structurally similar to those with more successful cognitive aging, without the structural or neurophysiological features of MCI or Alzheimer's disease, despite similarly poor cognitive function to MCI. Their underperformance on cognitive tests may be because of lower cognitive reserve and other risk factors across the lifespan.
