# Supplementary figures and images for: Neurophysiological and Brain Structural Markers of Cognitive Frailty Differ from Alzheimer's Disease
Source: J Neurosci. 2022 Feb 16;42(7):1362–73. doi: 10.1523/JNEUROSCI.0697-21.2021 (PMC8883844; doi:10.1523/JNEUROSCI.0697-21.2021)

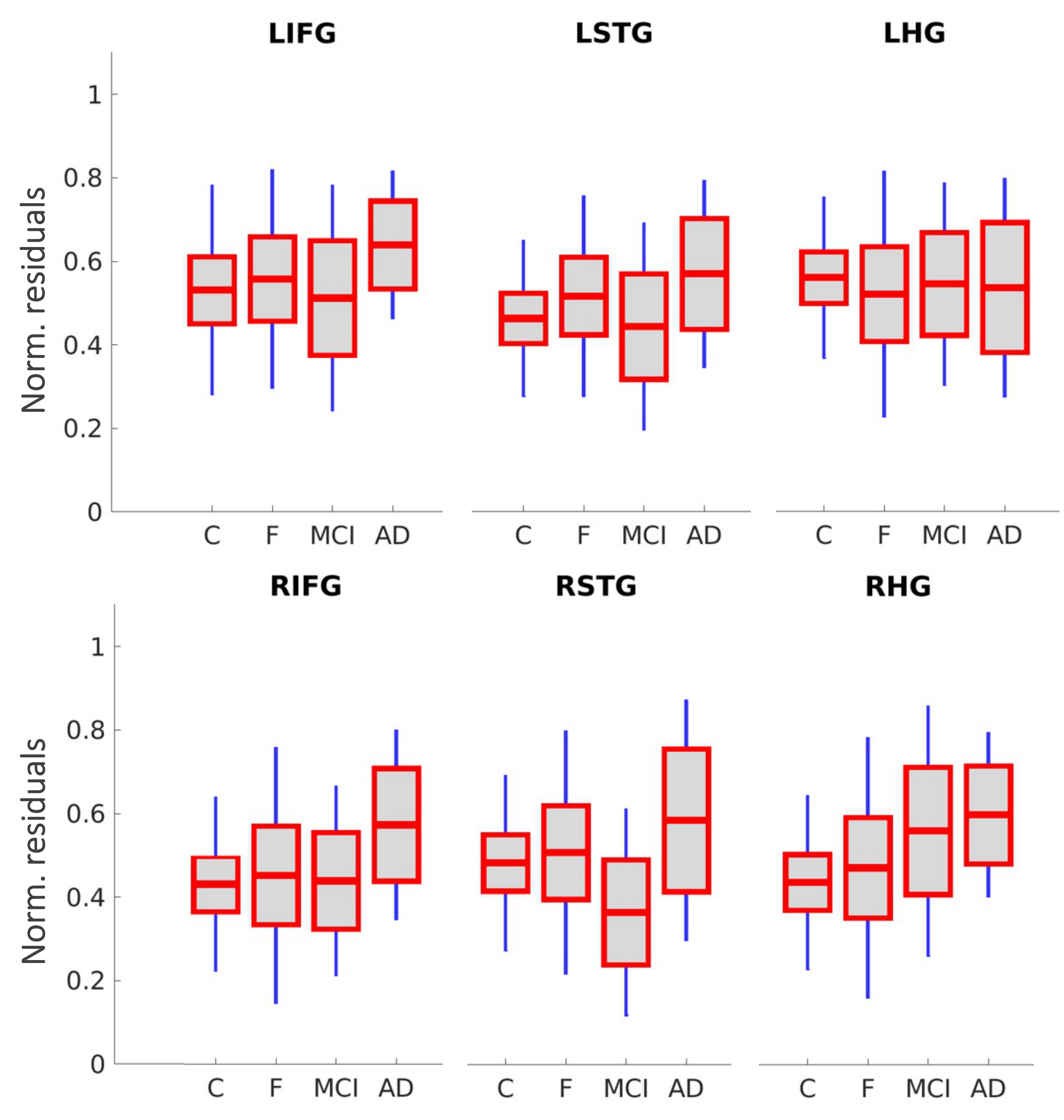

Supplement: Figure 2-1 — Gray matter volume analysis of the lateral frontotemporal network. In Alzheimer's disease. atrophy of the auditory cortex comes at the later isocortical Braak stages (i.e., V-VI) along with other neocortical areas such as the frontal cortex. Therefore, in the early stages of Alzheimer's disease, the integrity of the auditory cortex is not expected to be compromised, and consequently, diminished deviant responses observed in our clinical population are not expected be attributed to the atrophied auditory cortex. To provide support for this claim, we performed the confirmatory GMV comparisons across the four groups within the lateral frontotemporal ROIs which were used to extract neurophysiological signals from. We performed ANCOVAs to test for group differences while accounting for differences in age and TIV. As expected, there were no significant effect of the group in any of the six ROIs, confirming that the differences we find in the deviant responses could not be attributed to local GM atrophy. C, Controls; F, cognitively frail; AD, Alzheimer's disease. Download Figure 2-1, TIF file. [file ns-JN-RM-0697-21-s01.tif]

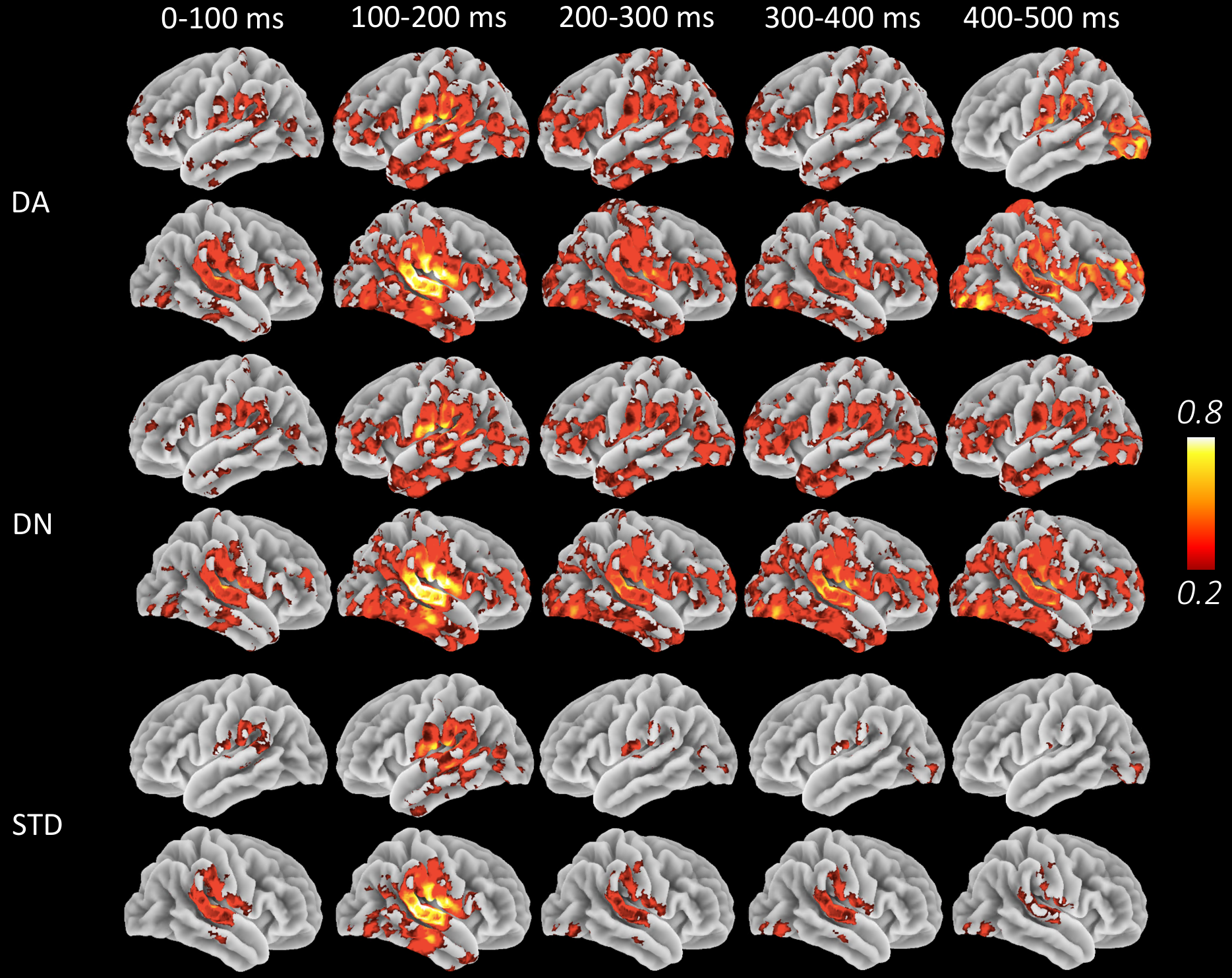

Supplement: Figure 3-1 — Source activity: The renderings display mean source activity in 100 ms moving time windows in the Control group. In line with the topoplot activity and our RMS findings, here we find the activation of a bilateral frontotemporal network peaking at 100 ms. Further, compared with the STD condition, deviant conditions show stronger and more widespread activity in the frontotemporal regions 200 ms after the sound onset. Download Figure 3-1, TIF file. [file ns-JN-RM-0697-21-s02.tif]
