# Supplementary material for: Neurophysiological and Brain Structural Markers of Cognitive Frailty Differ from Alzheimer's Disease
Source: J Neurosci. 2022 Feb 16;42(7):1362–73. doi: 10.1523/JNEUROSCI.0697-21.2021 (PMC8883844; doi:10.1523/JNEUROSCI.0697-21.2021)
Supplement: Extended Data Table 3-1 — Regional differences in the response to novelty deviants (DN) and associative deviants (DA) compared to standard trials. In this version of the analysis, we performed the statistical comparisons on the log transformed squared RMS time series, to ensure the normality assumption of the GLMs is met. The results of the RMS and log transformed RMS time series largely overlap. However in the latter analysis task-group interaction effects do not reach statistical significance. k: Cluster mass; pcor: Cluster corrected p-value. Download Table 3-1, DOCX file. [file ns-JN-RM-0697-21-s03.docx]

**Table 3-1.** Regional differences in the response to novelty deviants (DN) and associative deviants (DA) compared to standard trials. In this version of the analysis, we performed the statistical comparisons on the log transformed squared RMS time series, to ensure the normality assumption of the GLMs is met. The results of the RMS and log transformed RMS time series largely overlap. However in the latter analysis task-group interaction effects do not reach statistical significance. *k: Cluster mass; p_cor_: Cluster corrected p-value.*

| Contrast | ROI | Group | k | p_cor_ | Time |
| --- | --- | --- | --- | --- | --- |
| STD-DN | LIFG | Control | -497.43  -595.04 | 0.004  0.002 | 167-252  362-500 |
|  |  | Frail | -774,96 | 0.002 | 262-500 |
|  | RIFG | Control | -204.22 | 0.027 | 23-90 |
|  |  |  | -134.42 | 0.051 | 165-222 |
|  |  |  | -594.2 | 0.004 | 343-500 |
|  | LSTG | Control | -433.04 | 0.006 | 381-500 |
|  |  | Frail | -177.85 | 0.045 | 352-428 |
|  |  |  | -206.56 | 0.037 | 430-500 |
|  | RSTG | Control | -275.66 | 0.022 | 175-274 |
|  |  |  | -631.41 | 0.002 | 295-500 |
|  |  | Frail | -274.8 | 0.018 | 260-380 |
|  |  |  | -391.41 | 0.004 | 382-500 |
|  |  | Alzheimer | -182.9 | 0.041 | 438-500 |
|  | LHG | Controls | -273.65 | 0.024 | 388-500 |
|  | RHG | Controls | -336.9 | 0.01 | 329-487 |
|  |  | Frail | -138.69 | 0.043 | 1-48 |
| STD-DA | LIFG | MCI | -508.9 | 0.002 | 214-411 |
|  |  |  | -155.91 | 0.047 | 454-500 |
|  | RIFG | Control | -267.28 | 0.014 | 211-315 |
|  |  | Frail | -346.16 | 0.018 | 305-447 |
|  |  | Alzheimer | -775.37 | 0.008 | 177-500 |
